# Recurrence after percutaneous radiofrequency ablation of hepatocellular carcinoma: Analysis of the pattern and risk factors

**DOI:** 10.3389/fonc.2023.1018715

**Published:** 2023-02-23

**Authors:** Rui Chen, Beining Hou, Yanzhao Zhou, Tuo Zhang, Zhengzheng Wang, Xun Chen, Yingwei Zhang, Man Chen

**Affiliations:** ^1^ Department of Critical Care Medicine, Shandong Provincial Hospital affiliated to Shandong First Medical University, Jinan, China; ^2^ Faculty of Electronic Information and Electrical Engineering, Dalian University of Technology, Dalian, China; ^3^ Department of Hepatobiliary and Pancreatic Surgery, The Affiliated Cancer Hospital of Zhengzhou University & Henan Cancer Hospital, Zhengzhou, China; ^4^ Department of Critical Care Medicine, Shandong Provincial Hospital, Cheeloo College of Medicine, Shandong University, Jinan, China; ^5^ Beijing Key Laboratory of Mobile Computing and Pervasive Device, Institute of Computing Technology, Chinese Academy of Sciences, Beijing, China

**Keywords:** hepatocellular carcinoma (HCC), radiofrequency ablation (RFA), overall survival (OS), disease-free survival (DFS), prognosis, recurrence

## Abstract

**Background:**

Hepatocellular carcinoma (HCC) frequently relapses after minimally invasive treatment. This study aimed to observe the influencing factors of different recurrence patterns after radiofrequency ablation (RFA) for the treatment of recurrence.

**Methods:**

The medical records of HCC patients who underwent RFA between January 2010 and January 2019 were retrospectively reviewed. HCC recurrence is classified into three types: local tumour progression (LTP), intrahepatic distant metastasis, and extrahepatic metastasis. Risk factors, overall survival (OS), and disease-free survival (DFS) were assessed for each modality. Among the risk factors are age, gender, liver function tests, blood tests, and tumour size. The OS and DFS curves were measured by the Kaplan-Meier method.

**Results:**

406 patients who had undergone RFA were included in the study. The median survival for OS and DFS were 120 and 43.6 months. During follow-up, 39, 312, and 55 patients developed LTP, intrahepatic distant metastasis, and extrahepatic metastatic recurrence, respectively. The independent risk factors for each type were as follows: WBC > 5.55*10^9^/L was an independent risk factor for local recurrence. Multiple tumours, extrahepatic metastases, and AFP > 200 ng/ml were used for intrahepatic metastases. Age (*P* = 0.030), recurrence pattern (*P* < 0.001) and Child-Pugh class B (*P* = 0.015) were independent predictors of OS.

**Conclusions:**

According to our classification, each pattern of recurrence has different risk factors for recurrence, OS, and DFS.

## Introduction

Primary liver cancer (PLC) is a common cause of cancer and cancer-related death worldwide. Hepatocellular carcinoma (HCC) accounts for about 90% of PLC and is a major global health problem (1).

Radiofrequency ablation (RFA), a standard minimally invasive treatment modality, has been widely used in clinical practice for local control of liver tumours (2). Treatments such as RFA, transarterial chemotherapy, and radioembolization are the recommended treatments for unresectable HCC (3). For solitary, small HCC, there are studies combining RFA and surgical resection as the first-line treatment (1).

RFA is a safe and effective treatment for liver cancer. However, as with other local treatments, recurrence and metastasis after RFA remain a significant threat for liver cancer patients. Radiological methods, such as computed tomography (CT) or magnetic resonance imaging (MRI), are available, but surveillance programs will no longer be cost-effective. In general, it is not cost-effective to use multidetector CT or dynamic MR imaging for monitoring because of the high false-positive rate and the need for the use of contrast agents to achieve adequate sensitivity (4). Therefore, there is an urgent need for effective biomarkers, which can easily evaluate the recurrence mode and curative effect after RFA treatment. Moreover, HCC recurrence is classified into three types: local tumour progression (LTP), intrahepatic distant metastasis, and extrahepatic metastasis.

Recently, many studies have shown that the systemic inflammatory response is associated with tumor progression. Neutrophil-lymphocyte ratio (NLR) and platelet-lymphocyte ratio (PLR), which are inflammation-related biomarkers, have been confirmed to be prognostic indicators in gastric cancer (5, 6), colorectal cancer (7, 8) and Type A Acute Aortic Dissection (9). However, to our knowledge, few studies have reported the correlation between NLR, PLR, and different recurrence modes of RFA. Therefore, in this study, the value of inflammatory markers in predicting the prognosis of liver cancer patients receiving RFA was studied.

The aim of this retrospective study was to evaluate the long-term outcome of RFA alone as first-line treatment and to identify prognostic factors for different recurrence patterns in 406 patients. Each pattern of recurrence has different risk factors for recurrence, OS, and DFS.

## Materials and methods

### Patient characteristics

**Figure 1:**
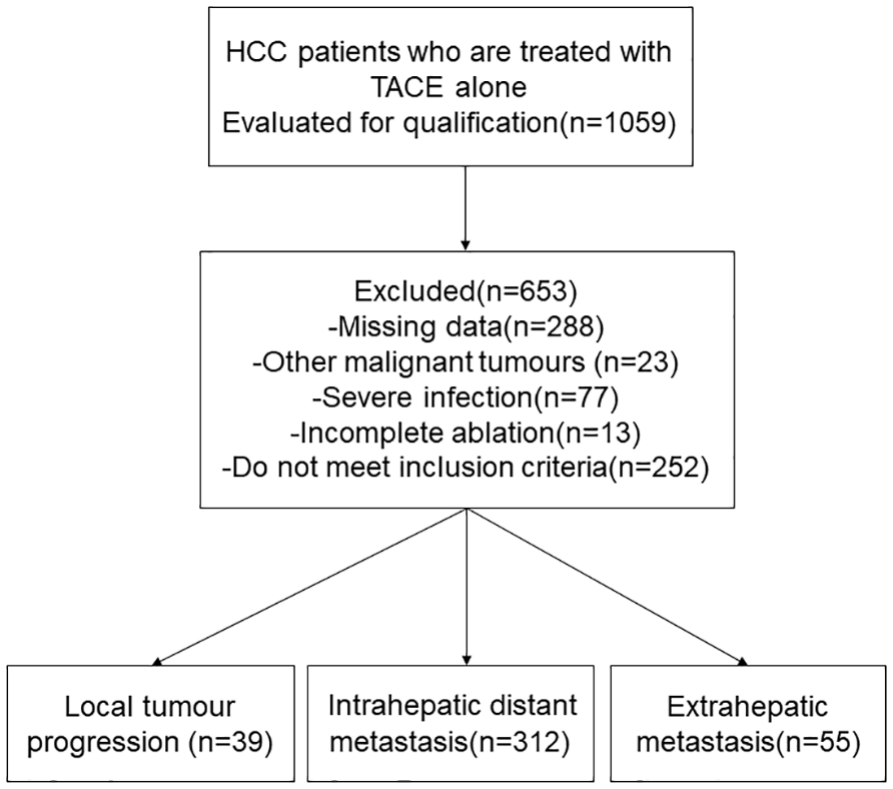
Flow chart shows the screening procedure for patients with hepatocellular carcinoma (HCC).

We retrospectively evaluated the data of 1059 patients with HCC who underwent RFA between January 2010 and January 2019 at the Affiliated Tumor Hospital of Zhengzhou University, China. The inclusion criteria for this study were: (1) Diagnosed as HCC(2) Single tumor, ≤ 5 cm in diameter;(3) There are multiple nodule (2-3 tumors, ≤ 3 cm in greatest diameter)(4) Patients with Child-Pugh class A or B and percutaneous RFA under the guidance of ultrasound in our hospital. The exclusion criteria for this study were: (1) other malignancies, (2) surgical contraindications, and (3) severe infection. Most patients were excluded due to a lack of complete case data. Ultimately, 406 patients with HCC treated with RFA were included in the study (**Figure 1**). Preoperative baseline data for all patients are shown in **Table 1**.

**Table 1:** Patient demographics (N= 406).

Characteristic for patients	N=406
Sex (Female/Male)	71 (17.5)/335 (82.5)
Age (year), Median (range)	59 (31-91)
Tumor diameter (cm), Median (range)	2.5 (0-5)
Tumor number, Median (range)	2 (1-3)
Cirrhosis type	
No	25 (6.2)
Hepatitis B	328 (80.8)
Hepatitis C	45 (11.1)
Alcoholic hepatitis	3 (0.7)
Others	5 (1.2)
Extrahepatic metastases (No/Yes)	392 (96.6)/14 (3.4)
Child-Pugh Class (A/B)	388 (95.6)/18 (4.4)
Pathological types	
Well-differentiated	29 (7.1)
Moderately differentiated	67 (16.5)
Poorly differentiated	18 (4.4)
No	292 (71.9)
Ascites (No/Yes)	381 (93.8)/25 (6.2)
ALT (U/L), Median (range)	25.6 (4-220.6)
AST (U/L), Median (range)	27.7 (11.7-408.4)
γ-GT (U/L), Median (range)	44.5 (10.2-1789.2)
AFP (ng/ml), Median (range)	15.6 (0.61-61100)
ALP (U/L), Median (range)	80 (11.4-439)
ALB (g/L), Median (range)	40 (23.4-68)
TBIL (μmol/L), Median (range)	14 (3.7-56.9)
Blood sugar, Median (range)	5 (3.6-78.5)
RBC count (×10^9^/L), Median (range)	4.3 (2-6.59)
Platelet count (×10^9^/L), Median (range)	103 (21-435)
Lymphocyte count (×10^9^/L), Median (range)	0.327 (0.046-76.5)
NLR	1.7 (0.007-19.7)
PLR	334.9 (2.6-2443.8)
WBC count (×10^9^/L), Median (range)	4.29 (1.1-11.49)
Neutrophil count (× 10^9^/L), Median (range)	0.571 (0.24-0.925)
INR	1.1 (0.82-1.82)
Ablation time (s), Median (range)	480 (120-2960)
Complication (No/Yes)	159 (39.2)/247 (60.8)
Recurrence pattern	
LPT	39 (9.6)
Intrahepatic distant metastasis	312 (76.8)
Extrahepatic metastatic	55 (13.5)

Continuous variables are expressed as the mean ± standard deviation, and categorical variables are expressed as N (%).

The values given are the number of patients unless indicated otherwise.

Our institutional review committee approved this retrospective study. As this article was a retrospective study, all patients gave up their written informed consent.

### Treatment instruments

The Samsung ACCUVIX A30 color Doppler ultrasound diagnostic instrument was used with an abdominal probe frequency of 3.5MHz. Radiofrequency ablation instrument the cold circulating radiofrequency ablation system produced by STAR med, Korea, consisted of a radiofrequency generator, cold circulating water pumps, negative polar plates, and accessories. The maximum output powerwas 200 W, stable control at 15-125°C, the ablation electrode needle ua sed cold circulating single motor ablation needleand, the length of the lead bare electrode was dynamically adjusted according to the specific tumor size (0-4 cm), the output power and ablation time were adjusted according to the lead bare length.

### Therapeutic method

The blood routine, coagulation function, and liver and kidney function were checked preoperatively, and enhanced CT or MRI examination was well-established. The puncture path was determined based on the specific location and size of the tumor under ultrasound. Ultrasound-guided live speaking ablation electrodes are needled into the tumor, and RFA begins after reaching the location. The specific ablation power and time were determined by the lesion size. Ultrasound during ablation was monitored in real-time to avoid damage to the intrahepatic ducts. Ablation margins for all tumors, except subcapsular, were planned to be at least 0.5 cm from the tumor border. The ablation process ends when the echogenic area created by RFA is large enough to cover the entire tumor and surrounding normal liver to achieve an adequate ablation margin on ultrasound.

The PLR is calculated as the platelet count divided by the lymphocyte count, and NLR is calculated as the neutrophils count divided by the lymphocyte count.

### Efficacy evaluation and follow-up

Dynamic contrast-enhanced CT or MRI was performed on the third day after RFA treatment to evaluate the treatment effect, and the complication status was recorded. Further treatment will be scheduled if any residual or new tumor is found. Imaging 1 month after treatment confirmed no definite activity of the tumor ablation zone as complete ablation. Meanwhile, we defined incomplete ablation as the presence of local enhancement or rim enhancement within the tumor lesion on dynamic contrast-enhanced CT or MRI of the liver on arterial phase examination. Meanwhile, patients underwent multiphase enhanced CT or MRI every 3 months for the first 2 years and every 4-6 months after that (10).

Local tumor progression(LPT) was defined as the recurrence of completely ablated tumors during follow-up (11). Intrahepatic distant recurrence is defined as the discovery of a new tumor in the liver that is not directly connected to the original lesion and is more than 1cm away. Extrahepatic metastases are defined as metastatic tumors in other organs outside the liver. Follow-up visits were conducted by telephone and outpatient revisits, laboratory and imaging findings were collected, and follow-up patient clinical data were recorded. All patients were followed up, and the date of completion of RFA therapy was the follow-up start date. We compared overall survival (OS) and disease-free survival (DFS) in patients with different recurrence patterns. OS is defined as the time from initial treatment to death or the last follow-up. DFS is defined as the proportion of patients who survive without recurrence and metastasis (disease-free) from the initial treatment.

### Statistical analysis

Continuous variables were expressed as mean ± standard deviation. The frequency (percentage) at which categorical variables are expressed. Univariate and multivariate Cox proportional hazards regression models were used to estimate hazard ratios (HR) and corresponding 95% confidential intervals (CI) for each potential prognostic variable. The OS and DFS curves were measured by the Kaplan Meier method. P values less than 0.05 were considered significant. Variables that were statistically significant (all P < 0.05) in univariate analysis and included in multivariate analysis. Analyses were performed using SPSS software version 25 (SPSS Inc., Chicago, IL) and R (version 4.1.2).

## Results

### Patient characteristics

Of the 406 patients with HCC, 335 (82.6%) were male and 71 (17.4%) were female. Their mean age was 59 years. LTP was detected in 39 of 406 patients after RFA, 312 cases of intrahepatic distant recurrence, and 55 cases of distant metastasis. Characteristics of recurrent tumors of each pattern were shown in **Table 2**. Other clinic characteristics, including tumor diameter, blood routine and liver function indexes, are shown in **Table 1**.

**Table 2:** Characteristics and outcomes for different recurrence patterns.

Characteristic for patients	LPT (N=39)	Intrahepatic distant metastasis (N=312)	Extrahepatic metastatic (N=55)
Sex (Female/Male)	11 (28.2)/28 (71.8)	50 (16.0)/262 (84.0)	10 (18.2)/45 (81.8)
Age (year), Median (range)	57 (38-83)	59.22 (31-91)	60.5 (34-83)
Tumor diameter (cm), Median (range)	3.3 (0-5)	2.3 (0.8-5)	2.7 (1.2-5)
Tumor number, Median (range)	2.2 (1-3)	2.4 (1-3)	1.6 (1-3)
Cirrhosis type			
No	4 (10.3)	16 (5.1)	5 (9.1)
Hepatitis B	34 (87.2)	247 (79.2)	47 (85.5)
Hepatitis C	1 (2.6)	41 (13.1)	3 (5.5)
Alcoholic hepatitis		3 (1.0)	
Others		5 (1.6)	
Extrahepatic metastases (No/Yes)	39 (100.0)/0 (0)	307 (98.4)/5 (1.6)	46 (83.6)/9 (16.4)
Child-Pugh Class (A/B)	36 (92.3)/3 (7.7)	298 (95.5)/14 (4.5)	54 (98.2)/1 (1.8)
Pathological types			
Well differentiated	1 (2.6)	23 (7.3)	5 (9.1)
Moderately differentiated	14 (35.9)	41 (13.1)	12 (21.8)
Poorly differentiated	1 (2.6)	16 (5.1)	1 (1.8)
No	23 (59.0)	232 (74.4)	37 (67.3)
Ascites (No/Yes)	34 (87.2)/5 (12.8)	294 (94.2)/18 (5.8)	53 (96.4)/2 (3.6)
ALT (U/L), Median (range)	24.5 (9.6-128)	26.4 (4-220.6)	24.8 (8.3-94.5)
AST (U/L), Median (range)	23.6 (14.1-129.8)	28 (11.7-408.4)	24.35 (12.9-110)
γ-GT (U/L), Median (range)	38.8 (14.6-257.2)	47.4 (11.3-1789.2)	39.8 (10.2-453.1)
AFP (ng/ml), Median (range)	26 (0.61-12134.6)	15.76 (0.81-61100)	11.98 (0.74-15729)
ALP (U/L), Median (range)	76.3 (26.2-161.8)	80.05 (11.4-439)	82.4 (39.6-242.7)
ALB (g/L), Median (range)	40.75 (24.3-46.5)	39.73 (23.4-68)	40 (28.8-48.8)
TBIL (μmol/L), Median (range)	14.4 (3.7-44)	14.125 (5.2-56.9)	12.6 (4.5-52.7)
Blood sugar, Median (range)	4.9 (4.19-12.19)	4.98 (3.61-78.5)	5.18 (3.71-14.49)
Hemoglobin (g/L), Median (range)	134 (67-171)	136.07 (68-178)	135.17 (103-163)
RBC count (×10^9^/L), Median (range)	4.36 (2.37-5.33)	4.26 (2.01-6.59)	4.315 (2.84-5.29)
Platelet count (×10^9^/L), Median (range)	127 (21-242)	100.33 (24-435)	117 (30-333)
Lymphocyte count (×10^9^/L), Median (range)	0.3157 (0.046-0.571)	0.329 (0.047-45.3)	0.305 (0.149—76.5)
NLR	1.83 (0.56-19.74)	1.71 (0.01-19.68)	1.83 (0.01-4.62)
PLR	411.59 (60.69-2237.62)	315.67 (2.60-2443.82)	379.56 (2.71-1558.66)
WBC count (×10^9^/L), Median (range)	4.9 (1.3-9.37)	4.22 (1.1-11.49)	4.61 (2.12-8.26)
Neutrophil count (× 10^9^/L), Median (range)	0.575 (0.317-0.908)	0.564 (0.257-0.925)	0.58 (0.24-0.743)
INR	1.1 (0.89-1.72)	1.11 (0.82-1.82)	1.077 (0.87-1.71)
Ablation time (s), Median (range)	600 (240-1740)	430 (120-2960)	462.86 (270-2280)
Complication (No/Yes)	16 (41.0)/23 (59.0)	122 (39.1)/190 (60.9)	21 (38.2)/34 (61.8)
Complete ablation (No/Yes)	0 (0)/39 (100.0)	3 (1.0)/309 (99.0)	0 (0)/55 (100.0)
Recurrence time (month)	8 (1-34)	10.29 (0-75)	10.5 (2-61)

Continuous variables are expressed as the mean ± standard deviation, and categorical variables are expressed as N (%).

The values given are the number of patients unless indicated otherwise.

### Risk factors of each recurrence pattern

**Table 3** shows the results of univariate and multivariate analysis of risk factors for each pattern of recurrence. Preoperative glucose > 4.5 mmol/L, HR [95% CI]: 0.055 [0.008, 0.378], *P* = 0.003) were protective factors for LTP, while preoperative white blood cell (WBC) > 5.55*10^9^/L (HR [95% CI]: 7.959 [1.719-36.850], *P* = 0.008) was an independent risk factor for LTP; Multiple tumors (HR [95% CI: 1.349 [1.035, 1.759], *P* = 0.027), extrahepatic metastases (HR [95% CI: 2.955 [1.205, 7.246], *P* = 0.018), and preoperative AFP level > 200 ng/ml (HR [95% CI: 1.475 [1.117, 1.948], *P* = 0.006) were independent risk factors for intrahepatic distant recurrence; Preoperative γ- GT > 74 ng/ml (HR [95% CI]: 0.415 [0.178 – 0.969], P = 0.042) was an independent protective factor for invasive recurrence.

**Table 3:** Univariate and multivariate analysis of the prognostic factors for different recurrence patterns in the entire study population (N = 406).

Factors	Univariate analysis		Multivariate analysis	
	HR (95% CI)	P value	HR (95% CI)	P value
LPT				
Sex		0.061		0.099
Male	1		-	
Female	3.074(0.951-9.932)		-	
Blood sugar (mmol/L)		0.018*		0.003*
≤4.5	1		1	
>4.5	0.140(0.027-0.717)		0.055(0.008-0.378)	
WBC count×10^9^/L		0.051*		0.008*
≤5.5	1		1	
>5.5	3.616(0.997-13.113)		7.959(1.719-36.850)	
Intrahepatic distant metastasis				
Tumor number		0.019*		0.027*
Solitary	1		1	
Multiple	1.373(1.054-1.789)		1.349(1.035-1.759)	
Extrahepatic metastatic		0.017*		0.018*
No	1		1	
Yes	2.970(1.215-7.262)		2.955(1.205-7.246)	
AFP (ng/ml)		0.003*		0.006*
≤200	1		1	
>200	1.526(1.157-2.011)		1.475(1.117-1.948)	
ALP (U/L)		0.025*		0.176
≤115	1		-	
>115	1.396(1.042-1.870)		-	
ALB (g/L)		0.020*		0.044
≤35	1		1	
>35	0.701(0.519-0.946)		0.733(0.542-0.991)	
RBC count×10^9^/L		0.047*		0.272
≤4.5	1		-	
>4.5	0.783(0.615-0.997)		-	
PLR		0.022*		0.054
<=390	1			
>390	1.329(1.041-1.697)			
Ablation time(s)		0.050		0.015*
<=430	1			
>430	0.794(0.631-1.000)		0.746(0.590-0.944)	
Extrahepatic metastatic				
γ-GT (U/L)		0.031*		0.042*
≤74	1		1	
>74	0.374(0.153-0.912)		0.415(0.178-0.969)	
NLR		0.048*		0.063
<=1.5	1		-	
>1.5	0.451(0.205-0.993)		-	

HR, Hazard ratio.

*Represent P< 0.05, which was considered to be statistically significant.

### OS and prognostic factors

**Figure 2:**
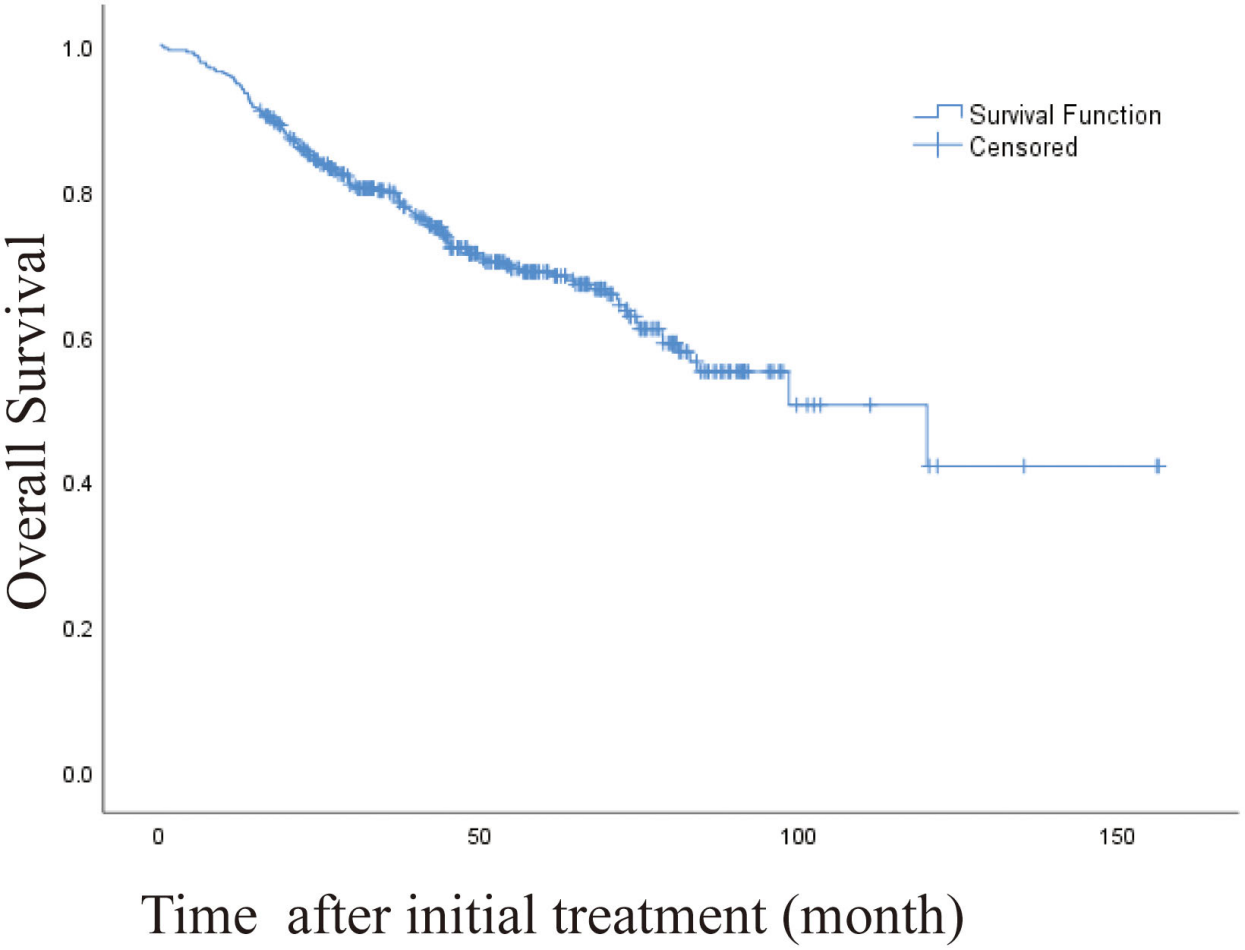
Kaplan-Meier curve of overall survival (OS) in patients with primary liver cancer who underwent radiofrequency ablation (median OS, 120 months).

**Figure 3:**
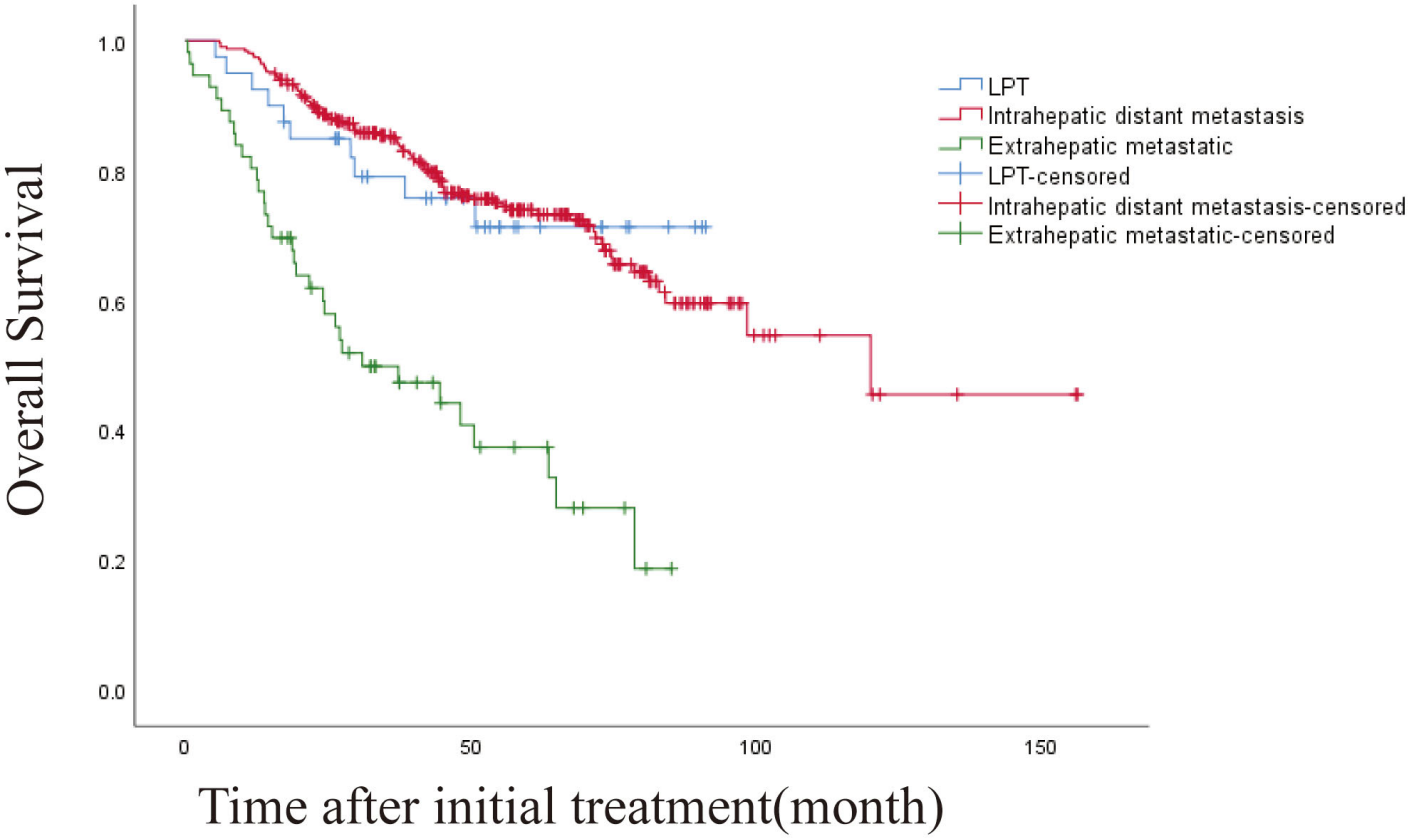
Kaplan-Meier curves of overall survival (OS) in patients with LPT, intrahepatic distant metastasis, and extrahepatic metastasis. (overall P<.001).

The 1 -, 3 -, and 5-year OS rates were 95.1%, 80.1%, and 69.2%, respectively. The median OS was 120 months (95% CI, 71.328-168.805 months) (**Figure 2**). Univariate and multivariate analysis showed that age > 60 years (HR [95%]: 1.487 [1.038-2.130], *P* = 0.030, Child-Pugh class B (HR [95%]: 2.247 [1.171-4.367], *P* = 0.015) and recurrence patterns of each pattern were observed: extrahepatic metastasis, 4.254 [2.026-8.931]; Overall P < 0. 001) were independent prognostic factors for OS (**Table 4**). The median OS was 120 months, 31 months (95% CI, 9.865-52.135), for patients with intrahepatic distant recurrence and extrahepatic metastases, respectively (**Figure 3**). However, the lowest survival rate for LPT was 70%, so the median survival time was not calculated.

**Table 4:** Univariate and multivariate analysis of the prognostic factors for overall survival in the entire study population (N = 406).

Factors	Univariate analysis		Multivariate analysis	
	HR (95% CI)	P value	HR (95% CI)	P value
Age (year)		0.006*		0.030*
≤60	1		1	
>60	1.638 (1.154-2.325)		1.487 (1.038-2.130)	
Child-Pugh Class		0.012*		0.015*
A	1		1	
B	2.94 (1.200-4.387)		2.247 (1.171-4.367)	
ALB (g/L)		0.019*		0.203
≤35	1		-	
>35	0.609 (0.403-0.923)		-	
Hemoglobin (g/L)		0.003*		0.513
≤143.5	1		-	
>143.5	0.537 (0.355-0.813)		-	
RBC count (×10^9^/L)		0.003*		0.006*
≤4.5	1		1	
>4.5	0.542 (0.360-0.816)		0.552 (0.360-0.845)	
Recurrence pattern		0.000*		0.000*
LPT	1		1	
Intrahepatic distant metastasis	1.061 (0.533-2.111)	0.866	0.982 (0.493-1.959)	0.960
Extrahepatic metastatic	4.052 (1.936-8.481)	0.000*	4.254 (2.026-8.931)	0.000*

HR, Hazard ratio.

*Represent P< 0.05, which was considered to be statistically significant.

### DFS and prognostic factors

**Figure 4:**
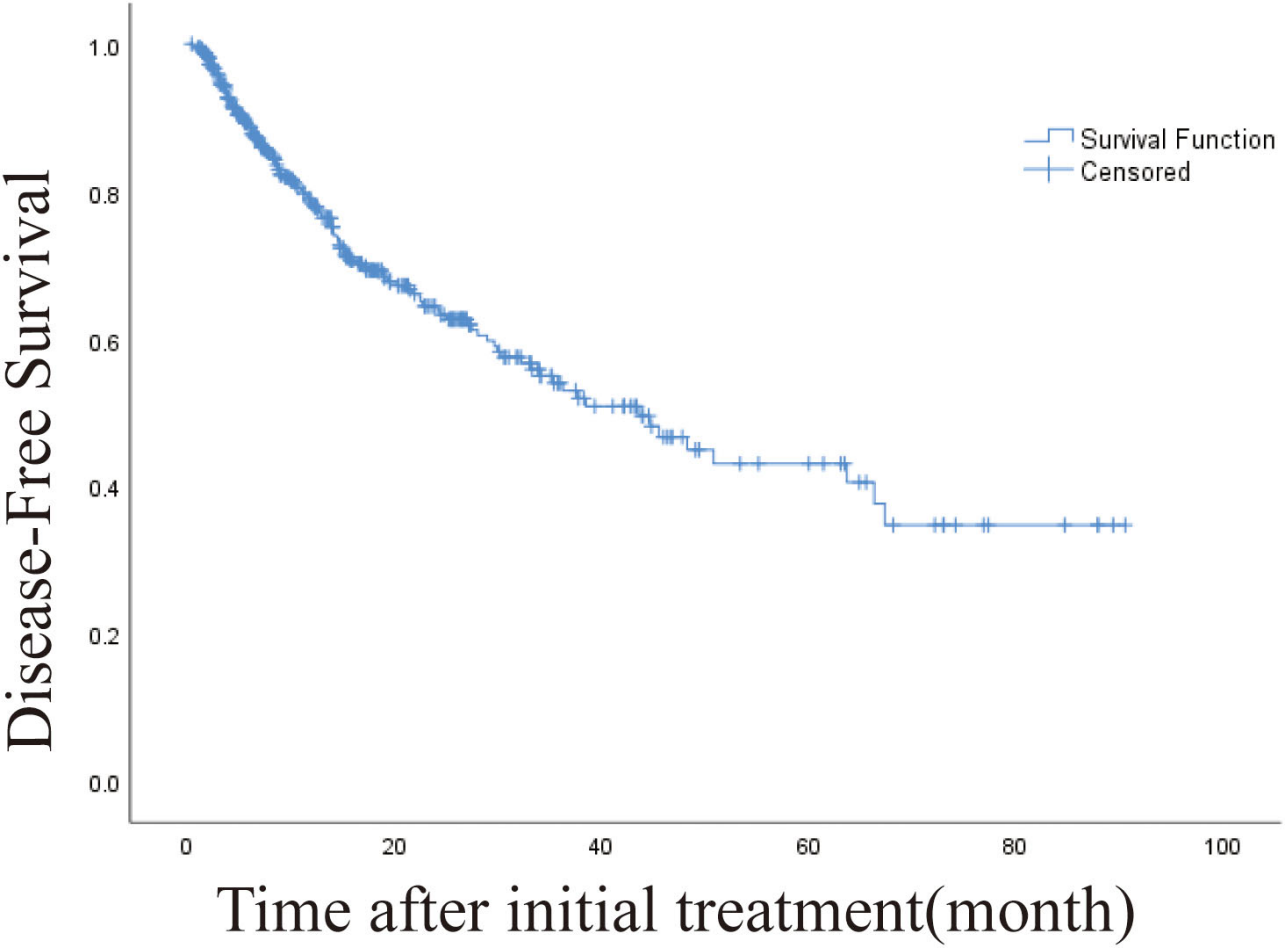
Kaplan-Meier curve of disease-free survival (DFS) in patients with primary liver cancer who underwent radiofrequency ablation (median DFS, 43.6 months).

**Figure 5:**
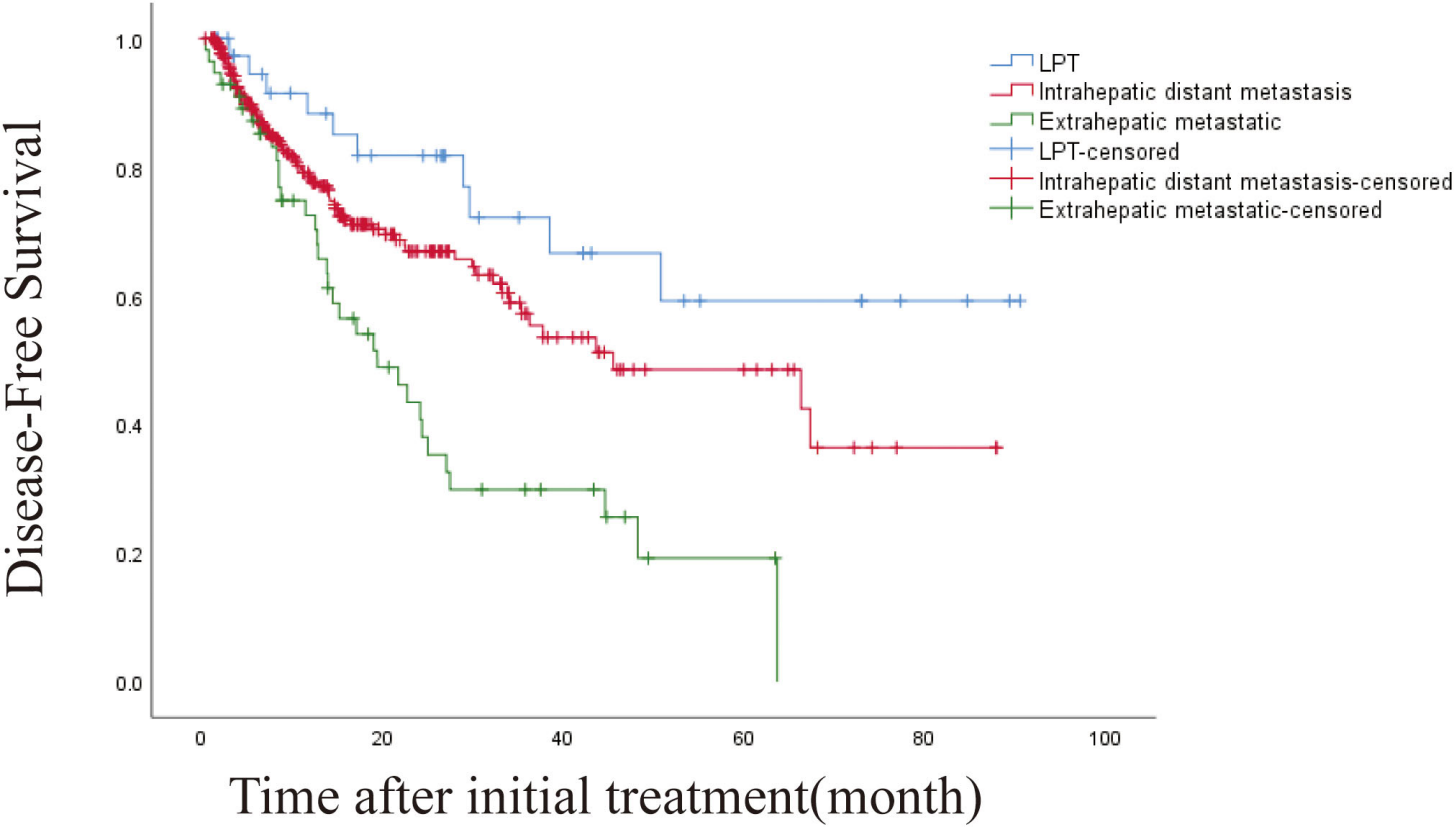
Kaplan-Meier curves of disease-free survival (DFS) in patients with LPT, intrahepatic distant metastasis, and extrahepatic metastasis.

The 1-, 3-, and 5-year DFS rates were 78.8%, 54.4% and 43.4%, respectively. The DFS of 406 patients with recurrence was 43.6 months (95% CI, 32.932-54.268 months). The DFS was 45.5 months (95% CI, 19.973-71.027) and 19.4 months (95% CI, 10.938-27.862) for intrahepatic distant metastasis and extrahepatic metastasis, respectively (**Figures 4**, **5**). Patients with intrahepatic distant metastases had a better median post recurrence survival than those with extrahepatic metastases (**Table 5**).

**Table 5:** Univariate and multivariate analysis of the prognostic factors for disease-free survival in the entire study population (N = 406).

Factors	Univariate analysis		Multivariate analysis	
	HR (95% CI)	P value	HR (95% CI)	P value
Age (year)		0.001*		0.024*
≤60	1		1	
>60	1.799 (1.264-2.559)		1.518 (1.057-2.179)	
Extrahepatic metastases		0.038*		0.403
No	1		-	
Yes	2.250 (1.048-4.830)		-	
Child-Pugh Class		0.032*		0.212
A	1		-	
B	2.033 (1.062-3.891)		-	
ALP (U/L)		0.030*		0.518
≤115	1		-	
>115	1.591 (1.045-2.422)		-	
ALB (g/L)		0.000*		0.004*
≤35	1		1	
>35	0.443 (0.291-0.672)		0.525 (0.338-0.815)	
Hemoglobin (g/L)		0.000*		0.875
≤143.5	1		-	
>143.5	0.474 (0.313-0.718)		-	
RBC count (×10^9^/L)		0.000*		0.007*
≤4.5	1		1	
>4.5	0.447 (0.296-0.676)		0.548 (0.354-0.849)	
Recurrence pattern				
LPT	1	0.000*	1	0.000*
Intrahepatic distant metastasis	2.050 (1.023-4.106)	0.043*	1.723 (0.855-3.471)	0.128
Extrahepatic metastatic	3.985 (1.890-8.401)	0.000*	3.761 (1.782-7.937)	0.001*

HR, Hazard ratio.

*Represent P< 0.05, which was considered to be statistically significant.

## Discussion

In this study, we evaluated RFA treatment risk factors for different recurrence patterns of HCC. In addition to a lower preoperative glucose level, a higher white blood cell count was a significant risk factor for LTP. Moreover, different recurrence patterns risk factors differ. We also observed 1 -, 3 -, 5 - overall survival rates of 94.9%, 80%, and 68.8% after undergoing RFA.

The link between cancer and inflammation has been studied for many years, and epidemiological studies have shown that chronic inflammation predisposes individuals to various types of cancer. Underlying infections and inflammatory responses are estimated to be associated with 15-20% of cancer deaths worldwide (12). This molecular pathway of cancer-related inflammation is currently being unraveled, leading to the discovery of novel target molecules and thus to improved diagnosis and treatment. Peripheral blood cells may reflect the inflammatory status of patients and the response of patients to malignancies, and these cells have great potential in improving the predictive power of known prognostic factors (13).

Lymphocytes are an essential immune cell in the inflammatory response and are independently associated with prognosis in various malignancies, such as gastric cancer (14), hepatocellular carcinoma (15), and lymphoma (16). Peripheral blood lymphocyte count is an important surrogate marker of immune reconstitution after stem cell transplantation for non-Hodgkin lymphoma, and lymphopenia is a surrogate marker of host immune insufficiency. Lymphopenia may compromise antibody-dependent cell-mediated cytotoxicity due to the lack of effector cells, thereby compromising the immune system’s efficacy (17). In addition, platelet tumor interactions have also been implicated in hematogenous metastasis, as antiplatelet agents and thrombocytopenia have been reported to reduce the number of experimental tumor metastases (18). In our study, PLR was shown to be an independent risk factor for distant intrahepatic metastasis in the univariate and multivariate analyses.

RFA is a representative method for nonsurgical treatment of liver tumors because of wide indications, minimal trauma, less bleeding, and rapid postoperative recovery (19). The study showed that RFA was safe and effective in treating liver tumors, and its efficacy was comparable to that of surgical resection (20). However, in previous studies, the 5-year recurrence rate still reached 65.6% - 69.8% (21), and a 5-year OS rate of 55. 2%-65. 1% (22). Our study classified liver cancer recurrence after RFA into: LTP, intrahepatic distant metastasis, and extrahepatic metastasis. The median OS and DFS showed significant differences among the patients. Intrahepatic and distant metastases were all associated with a longer median survival time than extrahepatic metastases. Furthermore, in univariate and multivariate analysis, we found that the recurrence pattern was an independent prognostic factor for OS, and each recurrence pattern had different risk factors. These results imply the usefulness of this classification for liver cancer recurrence, which provides us with an important clinical reference value for adopting preventive measures for each recurrence pattern according to these risk factors and helps us to predict the long-term survival of patients with recurrence. In our study, the 1-, 3-, and 5-year OS rates were 94.9%, 80% and 68.8%, respectively. The 1-, 3-, and 5-year DFS rates were 78.3%, 54% and 43.1%, respectively.

According to the literature, due to the risk of liver function deterioration, Child-Pugh class B patients should selectively consider RFA (23). Our study also found that Child-Pugh class B was another independent predictor consistent with previous findings (24). Child-Pugh class B liver disease may result in intolerance to repeated rescue therapy and even liver failure at long-term follow-up. Local tumor recurrence after RFA is closely related to the location of the tumor, such as the tumor located in the top of the diaphragm, adjacent to the great vessels, and adjacent to the gallbladder (25, 26). In addition, our study found that AFP level was an independent predictor of distant intrahepatic metastasis ≥ 200 ng/ml. Many tumor markers have been investigated, including AFP, AFP-L3, and DCP. Biomarkers are also crucial in diagnosing, predicting prognosis, and surveillance of liver cancer (27, 28). High levels of AFP indicate poor differentiation and high invasiveness of initial HCC, which are also associated with tumor progression after treatment (29). Liver function is also an important measure of the quality of patient survival. Liver function, viral load, and adverse effects were examined (30) and sequential cellular immunotherapy improves progression-free survival for patients with HCC in Cui’s study. In addition, the purpose of the meta-analysis of Giuseppe et al. was to evaluate the recurrence and survival probability of HCV related diseases, potential cure, early HCC with complete remission after treatment, and to identify predictors of recurrence and survival (31). In our study, liver function served as an independent prognostic factor in intrahepatic distant metastasis and extrahepatic metastatic recurrence. Men dominate PLC, and the proportion of men and women is estimated to be 2-2.5:1 (32). In our study, male sex was equally represented and acted as an independent prognostic risk factor for LPT.

Our study has several limitations: First, it is a single center retrospective study and therefore inherently subject to selection and indication biases; Second, the factors affecting prognosis are numerous, and those of our statistics are limited. The conclusions of this study need to be validated by prospective studies with larger sample sizes.

## Conclusions

In conclusion, the recurrence pattern of HCC after RFA treatment can be divided into LTP, intrahepatic distant metastasis, and extrahepatic metastasis. For different recurrence patterns, there are different risk factors, OS and DFS.

## Data availability statement

The original contributions presented in the study are included in the article/supplementary material. Further inquiries can be directed to the corresponding authors.

## Ethics statement

The studies involving human participants were reviewed and approved by the Ethics Committee of the Affiliated Tumor Hospital of Zhengzhou University, China.

## Author contributions

Software, formal analysis, investigation, and writing of the original draft (RC, BNH,YZZ). Acquisition of the data (TZ, ZZW). Analysis and interpretation of data (XC). Drafted the manuscript (RC). Critical revision of the manuscript for important intellectual content (YWZ). Study supervision (MC). All authors reviewed and commented on the manuscript and approved the final version.
